# Effects of genotype, sex, and feed restriction on the biochemical composition of chicken preen gland secretions and their implications for commercial poultry production

**DOI:** 10.1093/jas/skac411

**Published:** 2022-12-22

**Authors:** Veronika Gvoždíková Javůrková, Petr Doležal, Adéla Fraňková, Monika Horák, Darina Chodová, Iva Langrová, Eva Tůmová

**Affiliations:** Institute of Vertebrate Biology of the Czech Academy of Sciences, 603 65 Brno, Czech Republic; Groningen Institute for Evolutionary Life Sciences, University of Groningen, 9747 AG, Groningen, The Netherlands; Department of Animal Science, Faculty of Agrobiology, Food and Natural Resources, Czech University of Life Sciences, Prague 6 - Suchdol, Czech Republic; Department of Agroenvironmental Chemistry and Plant Nutrition, Faculty of Agrobiology, Food and Natural Resources, Czech University of Life Sciences, Prague 6 - Suchdol, Czech Republic; Department of Food Science, Faculty of Agrobiology, Food and Natural Resources, Czech University of Life Sciences, Prague 6 - Suchdol, Czech Republic; Department of Animal Science, Faculty of Agrobiology, Food and Natural Resources, Czech University of Life Sciences, Prague 6 - Suchdol, Czech Republic; Department of Animal Science, Faculty of Agrobiology, Food and Natural Resources, Czech University of Life Sciences, Prague 6 - Suchdol, Czech Republic; Department of Zoology and Fisheries, Faculty of Agrobiology, Food and Natural Resources, Czech University of Life Sciences, Prague 6 - Suchdol, Czech Republic; Department of Animal Science, Faculty of Agrobiology, Food and Natural Resources, Czech University of Life Sciences, Prague 6 - Suchdol, Czech Republic

**Keywords:** antimicrobials, ectoparasites, feather condition, chemosignaling, uropygial gland, welfare

## Abstract

Preen gland secretions spread on the feathers contain various chemical compounds dominated by fatty acids (**FAs**) and volatile organic compounds (**VOCs**). These chemicals may significantly affect plumage condition, microbial and ectoparasitic load on feathers, and chemical communication of birds. However, how chemical composition of preen secretions varies in commercially produced chickens with respect to their genotype, sex, and feeding regime remain largely unknown, as well as the welfare implications for farmed poultry. We found that while polyunsaturated fatty acids in chicken preen secretions differed significantly with genotype (*P* << 0.001), saturated fatty acids and monounsaturated fatty acids varied with genotype-dependent preen gland volume (*P* < 0.01). Chickens of meat-type fast-growing Ross 308 genotype had reduced preen gland volume and lower proportions of all FA categories in their preen secretions compared with dual-purpose slow-growing ISA Dual chickens. A total of 34 FAs and 77 VOCs with tens of unique FAs were detected in preen secretions of both genotypes. While differences in the relative proportion of 6 of the 10 most dominant VOCs in chicken preen gland secretions were related to genotype (*P* < 0.001), only 1 of the 10 most dominant VOCs showed a sex effect (*P* < 0.01), and only 2 of the 10 most dominant VOCs showed a genotype-dependent effect of feed restriction (*P* < 0.05). Feed restriction had no effect on the relative proportion of any of the FAs in chicken preen gland secretions. Moreover, we found that meat-type Ross 308 preen secretions were dominated by VOCs, which are proven attractants for poultry red mite and may also increase infestation with other ectoparasites and negatively influence overall odor-mediated intraspecific communication and welfare. This study shows that no feeding management, but long-term genetic selection in commercial breeding may be the main cause of the differences in the biochemistry and function of chicken preen secretions. This might have negative consequences for chemosignaling, antiparasitic, and antimicrobial potential of preen secretions and can lead to increased susceptibility to ectoparasites, plumage care disorders, and can affect the overall condition, welfare, and productivity of commercially bred chickens. Selection-induced preen gland impairments must therefore be considered and compensated by proper management of the chicken farm and increased care about animal well-being.

## Teaser

This study provides the first evidence for the role of genetic selection, preen gland volume, and their interaction on differences in the complex biochemistry of preen secretions and evaluates the implications for chemosignalling, antiparasitic, and antimicrobial function of preen secretions in commercially reared chickens.

## Introduction

The preen gland is the main and largest organ of sebum production in birds and is found in most species including poultry (Stettenheim, 2000). The preen gland produces an oily secretion that is spread over the entire surface of the body by the beak during the feather-cleaning activity called preening. Preening is an important part of the birds’ comfort behavior, comprising approximately 10% to 15% of their overall daily activity (Delius, 1988). However, whereas laying hens preen their feathers only twice a day in short periods (Vanliere et al., 1991), slow- and fast-growing broiler chickens spend on average 6.3% and 9% of their total daily activity with preening, respectively (Bokkers and Koene, 2003). Thus, feather greasing with preen secretions appears to be an important activity not only for wild birds but also for commercially produced chickens, which most probably also benefit from the protective functions of the preen gland secretions.

The preen gland secretion is a chemically complex mixture of lipids, wax esters, hydrocarbons, triglycerides, sterols, free fatty acids, alcohols, and volatile organic compounds (Jacob, 1975; Jacob and Ziswiler, 1982; Soini et al., 2007; Karlsson et al., 2010). In domestic chickens, wax diesters account for 49% of all lipids secreted from the preen gland (Wertz et al., 1986). The other dominant components are triacylglycerols (31.7%) and phospholipids (13.3%; Wertz et al., 1986) In any case, preen secretions are a unique source of rare lipids that are different from other types of lipids, such as storage fats, and that are not found elsewhere in the body (Saito and Gamo, 1968). These lipids are likely to protect feathers from fraying, mechanical abrasion, and water wetting, which is particularly important in waterfowl (Jacob and Ziswiler, 1982). However, the role and importance of individual substances in the secretions of the preen glands vary considerably (Moreno-Rueda, 2017). Lipids, hydrocarbons, and some peptides present in secretions have been shown to have antifungal effects and may help to protect against skin diseases (e.g., mycoses; Bandyopadhyay and Bhattacharyya, 1999). The antimicrobial effects of preen gland secretions have also been demonstrated in vitro (Shawkey et al., 2003; Ruiz-Rodriguez et al., 2009; Giraudeau et al., 2013; Verea et al., 2017; Braun et al., 2018; Alt et al., 2020), in vivo (Moller et al., 2009; Leclaire et al., 2014a; Bodawatta et al., 2020), and on wild birds (Jacob et al., 2014; Fulop et al., 2016; Giraudeau et al., 2017). Similarly, several experimental studies have documented that removal of the preen gland led to significant changes in feather bacterial composition (Bandyopadhyay and Bhattacharyya, 1996; Czirjak et al., 2013). Preen secretions are also rich in volatile organic compounds (**VOCs**; Soini et al., 2007, 2013; Haribal et al., 2009; Whittaker et al., 2010), which are important semiochemicals (Campagna et al., 2012). Many bird species have a highly developed sense of smell (Steiger et al., 2008). This is also true for the domestic chicken, whose genome contains at least 229 genes for olfactory receptors (Lagerstrom et al., 2006). Unsurprisingly, there is considerable evidence for the role of preen secretions in chemical communication between individuals, facilitating species recognition (Gebauer et al., 2004; Zhang et al., 2009; Gabirot et al., 2016), sex recognition (Whittaker et al., 2010; Zhang et al., 2010; Leclaire, et al., 2011; Amo et al., 2012), sexual mate choice and kin recognition (Whittaker et al., 2011; Bonadonna and Sanz-Aguilar, 2012; Leclaire et al., 2014b; Potier et al., 2018), or even individual recognition, with young goslings of the domestic goose (*Anser anser f. domestica*, Kerr, 1792) being able to distinguish the scent of their mother’s secretions from other geese (Wurdinger, 1982). This semiochemical function of preen gland secretions is already used in commercial breeding practice in the form of MHUSA (Mother Hens’ Uropygial Secretion Analogue), which is a synthetic analog of preen (syn. uropygial) secretion (Pageat, 2010). The application of MHUSA reduces stress and improves performance, physiological, and behavioral parameters during broiler chick production (Madec et al., 2008; Asproni et al., 2020). Finally, some VOCs in preen secretions such as carboxylic acids and aldehydes may also have a repellent effect against ectoparasites and mosquitos (Hwang, et al., 1982; Douglas et al., 2001, 2004, 2005).

The chemical composition of poultry preen secretions can be influenced by several factors. These include inter-individual variability (Karlsson et al., 2010), age (Kolattukudy and Sawaya, 1974), sex (Jacob et al., 1979), season (Kolattukudy et al., 1987; Fischer et al., 2017), or diet (Apandi and Edwards, 1964). However, it should be noted that in general very little attention has been paid to the study of factors affecting the chemical composition of preen secretions in birds. Moreover, broiler chickens are under constant selection pressure to increase performance and/or meat quality through feed conversion manipulation. Despite these facts and the apparently essential functions of the preen gland and its secretions in poultry, there is a lack of studies that have experimentally evaluated the factors influencing the composition of preen gland secretions and inferred their implications for poultry production and welfare.

The aim of this study was to describe in detail the chemical composition of the preen secretions of two chicken genotypes, fast-growing meat-type Ross 308 and dual-purpose slow-growing ISA Dual, with a particular focus on FAs and VOCs, and to experimentally test the effect of sex and feed restriction on the FAs and VOCs profiles in the preen secretions of these two chicken genotypes. The findings were then compared with available literature and implications for commercial poultry production and welfare were discussed.

## Material and Methods

### Ethical statement

The experiment was approved by the IACUC—Ethical Committee of the Central Commission for Animal Welfare of the Ministry of Agriculture of the Czech Republic and carried out in accordance with Directive 2010/63/EU for animal experiments (https://www.eurofawc.com/home/16).

### Animals and experimental design

In this study, meat-type fast-growing genotype Ross 308 and the dual-purpose slow-growing genotype ISA Dual were used. A total of 960-d-old chicks (male:female ratio, 1:1) were hatched at the International Test Station Ústrašice in the Czech Republic under standardized conditions. Chicks were weighed, wing banded, and randomly assigned to eight experimental groups based on a 2 × 2 factorial design for each genotype (i.e., for each genotype (ISA Dual and Ross 308), there were four experimental groups differing in sex (M:F) and two types of feeding regimes: 1) chickens fed ad libitum and 2) chickens with 30% restriction in the amount of feed between 14 and 21 d of age (i.e., chicks received only 70% of the amount of feed consumed by the ad libitum group). The amount of feed for the restricted groups was calculated daily based on the feed intake of the ad libitum groups. Chickens in the restricted groups were fed ad libitum before and after feed restriction period. Water was available ad libitum for all birds during the entire experiment. Each experimental group (*n* = 8) consisted of three replicates resulting in total of 24 littered poultry pens with 40 birds per pen (i.e., 14 birds per m^2^). During the experiment, three-phase feeding was used as it was previously described in Tumova et al. (2021). The starter phase was from 1 to 14 d. The grower phase in Ross 308 chickens was between 15 and 25 d and ISA Dual chickens between 15 and 35 d. The finisher phase in Ross 308 chickens was from 26 to 31 d and in ISA Dual chickens it was from 36 to 80 d of age. The starter feed contained 216 g/kg crude protein (**CP**) and 12.5 MJ ME, grower 196 g/kg CP and 12.9 MJ ME, and finisher 185 g/kg CP and 13.5 MJ ME. The lighting regime consisted of 23 h of light on days 1 to 7 and 18 h of light from day 8 to the end of the experiment.

The experiment was terminated when the chicks of each genotype reached a live weight of approximately 2,000 g. Four birds were then randomly selected from each pen (i.e., 12 chicks per group) for subsequent preen secretions analyses. The fast-growing Ross 308 chicks reached the expected slaughter weight of 2,000 g at 31 d of age and the slow-growing ISA Dual chicks at 80 d of age.

### Sampling of preen gland secretions

Experimental chickens were slaughtered in the experimental slaughterhouse of the International Poultry Testing Station Ústrašice in the Czech Republic by electrical stunning and bleeding from the jugular vein. Then, chicks were de-feathered, quickly washed with hot water, and weighed on a Kern EMB 1000-2 laboratory scale. The size of the preen gland (length × width × height) was then measured using a digital caliper with an accuracy of 0.01 mm and used for calculation of preen gland volume (Magallanes et al., 2016) which corresponds to preen gland secretory potential (Moreno-Rueda, 2017). The preen secretion was then manually squeezed and collected into 2.0 mL Eppendorf Safe-Lock tubes and stored at −20 °C until FAs and VOCs analyses. All sampling and handling were performed with rubber gloves.

### FAs analysis

#### Derivatization of preen gland secretion FAs

The samples of preen secretions were processed by alkaline transmethylation. First, 1 to 2 mg of preen secretions were dissolved in 1 mL of petroleum ether (Penta, Prague, CZ) with the help of an ultrasound bath. Subsequently, the sample was transferred to the 10 mL volumetric flask containing 1 mL of 0.4 M NaH (Sigma Aldrich, CZ) dissolved in methanol (VWR Chemicals, CZ). The flask was mixed, closed with the cap, and let to stand for 20 min at room temperature. After that, distilled water was added to the sample, and the flask was thoroughly mixed again. Finally, an aliquot of the separated organic layer was transferred to the vial and stored in the refrigerator until further analysis.

#### Identification and relative quantification of FAs by gas chromatography

A gas chromatograph GC 7890A coupled with a quadrupole MS 5975C (both Agilent, USA) was used for the identification of the FA profile in preen secretions. The GC was equipped with a fused silica column Rt-2560 (length 100 m, i.d. 250 μm, d.f. 0.2 μm, Restek, USA). The sample (1 μL) was injected at split mode (1:50) at an injector temperature 225 °C. The oven temperature started at 70 °C, which was held for 2 min, then increased at a rate of 5 °C/min to 225 °C and was held for 9 min. Finally, it was raised at a rate of 10 °C/min to 240 °C and held for 6.5 min (total run time 50 min). The mass detector operated in scan mode in a range of 40 to 400 Da. The ion source and quadrupole temperatures were set at 230 °C and 150 °C, respectively. FAs were identified by comparing their retention times and mass spectra with the retention times and mass spectra of available standards (FAME mix, Sigma Aldrich, CZ), and by comparing the data with the NIST database, version 2.0. A gas chromatograph GC 7890A with flame ionization detector (**FID**) was used for the relative quantification of FAs. The chromatographic conditions of the analysis were the same as presented above. The FID detector was heated to 260 °C, the hydrogen gas flow was set to 30 mL/min, the airflow at 400 mL/min, and makeup nitrogen flow was 30 mL/min. All samples were measured in triplicate.

### VOCs analysis

Headspace solid-phase micro-extraction coupled to multidimensional gas chromatography/mass spectrometry was used for analysis of VOCs in chicken preen secretions. All the samples processed in 1 d were thawed at the laboratory temperature (23 °C) and weighed using lab scale with a precision of 0.1 mg. The average weight of each sample was 27.5 ± 2.5 (mean ± SE) mg. In some cases, the amount was smaller with the average weight of preen gland oil 10 ± 2.5 mg. The samples were transferred in the 5 mL headspace (**HS**) vials and spiked with 10 µL of internal standard—2,6-dichloroanisole, purchased from Merck (Germany), diluted in ultra-pure water at concentration of 1 µg/mL. Prepared vials were put in the water bath and incubated for 3.5 h at 45 °C. After this, the 50 μm divinylbenzene/carboxen/polydimethylsiloxane (Supelco, Bellefonte, PA, USA) solid phase microextraction (**SPME**) fiber was exposed to the HS above the sample in the vial. This extraction process was optimized in a pre-experiment where different SPME fibers, extraction temperatures, and equilibration times were tested (see Supplementary Methods S1 for details). The fiber was sorbed in vials for 40 min at 45 °C, and then the analytes were desorbed for 7 min at 260 °C in the injection port of a multidimensional gas chromatography/mass spectrometry GCMS-QP2010 Ultra Shimadzu (Kyoto, Japan). This analytical system works with two columns in two ovens (GC1 and GC2) with different types of stationary phases. In the GC1, a fused-silica capillary SLB-5ms column (length 30 m, i.d. 0.32 mm, 1.0 μm d.f., Supelco, USA) was utilized. The SPB-50 column (length 30 m, i.d. 0.32 mm, 0.25 μm d.f., Sigma-Aldrich, Merck, Germany) was installed in the GC2. An inlet (with SPME-liner) was heated at 280 °C during the whole analysis. Between GC1 and GC2, “Deans switching” device (SHIMADZU 221-71468-91 Switching Assay, Shimadzu, Kyoto, Japan) was installed which switched completely chromatogram from GC1 to GC2 with 100% switching recovery (“Heart-cutting”). The switching pressure was set at 50 kPa. The switching time was set from 4 to 43 min. Helium was used as carrier gas (5.0 grade) with a constant flow rate of 80 kPa (approx. 1.37 mL/min) at the head of the column. The 45 min GC method began with an initial oven temperature of 45 °C for 5 min, followed by a ramp of 10 °C/min to 250 °C, and ending with a 17.5 min hold. In GC2, the same temperature program was applied. The transfer line between the GC2 and MS was heated at 280 °C. The ion source and MS transfer-line temperatures were set at 230 °C and 260 °C, respectively. The quadrupole mass analyzer was operated in electron ionization mode and scanned over a mass range of m/z 50 to 550 in full scan mode, having a filament bias voltage of −70 eV. To control this system, the software MDGC Solution 1.01.00 and GC Solution 240.00 were utilized. The mass spectra of the assigned chromatographic peaks were compared with those in the NIST 11 library (Stein, 1999) and interpreted. For the Van den Dool and Kratz linear retention index determination of compounds, C7–C40 saturated alkane mix in hexane (Supelco, Madrid, Spain) was repeatedly analyzed under the same experimental conditions. These computed retention indices of preliminarily identified compounds were compared with those obtained from the literature.

### Statistical analysis

Linear mixed-effects models (**LMMs**) were used to analyze the data, and the identity of the experimental replicate (i.e., the littered poultry pen) was included as a random effect in all models to account for this source of variability.

First, a separate LMM was used to test the role of sex, genotype, feeding restriction, and their two-way interactions on differences in preen gland volume (mm^3^) calculated from gland length, width, and height (Magallanes et al., 2016). Total body weight was not included in the LMM because no correlation was found between preen gland volume and total body weight (Spearman’s rank correlation: S = 79,944, *ρ* = −0.011, *P*-value = 0.9242).

Three separate LMMs were used to assess the role of genotype, sex, feed restriction, total body weight, and preen gland volume, including their two-way interactions and a three-way interaction between genotype, sex, and preen gland volume on the relative proportion of saturated fatty acids (**SFAs**), polyunsaturated fatty acids (**PUFAs**) and monounsaturated fatty acids (**MUFAs**) in chicken preen secretions. The relative proportions of MUFAs were log-transformed to achieve normality.

Ten separate LMMs were used to assess the role of genotype, sex, feed restriction, total body weight, and preen gland volume, including their two-way interactions on the relative proportion of the 10 most dominant VOCs in chicken preen secretions. The logarithmic transformation for 1-penten-3-ol, 3-ethyl-3-methylheptane, and hexanal and the square root transformation for the seven remaining VOCs were used to achieve normality of the VOCs proportional data.

Analyses were performed in R software (version 4.0.4, R; R Core Team, 2020) running on RStudio 1.1.453 (RStudio Team, 2015) using *lme4* (Bates et al., 2015), *lmerTest* (Kuznetsova et al., 2017), *ggplot2* (Wickham, 2016), and *ggpubr* (Kassambara, 2020) packages. Instead of a method using significance tests based on stepwise procedures, the results of LMMs in which all nonsignificant interactions were removed in one step and thus containing only main effects including insignificant and all significant interactions are presented as the statistically most robust models (Mundry and Nunn, 2009). Results of full LMMs are provided in Supplementary Tables S3, S4A–C, and S5 A–I. Goodness of fit for each model was tested using the r package *performance* (Lüdecke et al., 2021) and their graphical representation is shown in Supplementary Figure S1. Tukey post hoc tests based on Kenward–Roger degrees of freedom method were used for multiple comparison of significant effects and their means between tested categories using r package *lsmeans* (Lenth, 2016).

## Results

### Factors affecting chicken preen gland volume

**Figure 1.**
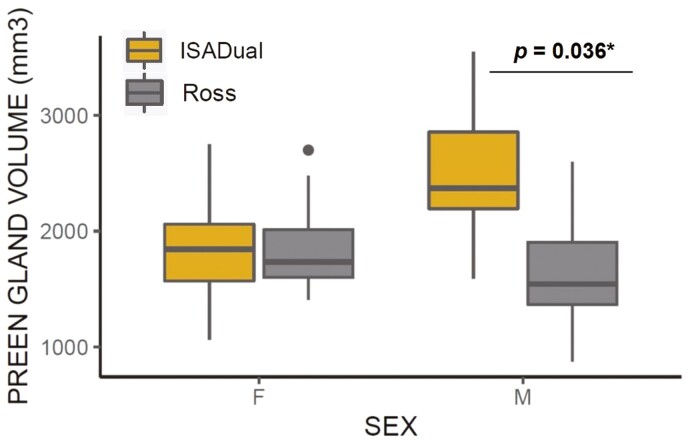
Interactive effect of genotype and sex on different secretory potential of chicken preen gland expressed as preen gland volume. Significant differences are based on Tukey post hoc test.

Of all the variables tested, genotype (*P* < 0.001), and interaction between genotype and sex (*P* << 0.001) significantly affected the preen gland secretory potential expressed by the preen gland volume (Table 1). While ISA Dual cockerels showed significantly larger preen gland volume compared to Ross 308 cockerels (Tukey post hoc test: *P* = 0.036; Figure 1), this difference was not evident in females of different genotypes (Tukey post hoc test: *P* = 0.998; Figure 1). Difference in preen gland volume between ISA Dual males and females only tend to be significant (Tukey post hoc test: *P* = 0.069; Figure 1). Feed restriction, total body weight and sex alone, and their interaction had no effect on preen gland volume (Table 1).

**Table 1.** LMM results evaluating the effect of genotype, feed restriction, sex, and their two-way interactions controlled for random effect of experimental pen on the chicken preen gland volume (*N* = 78)

Explanatory variables	df	*χ* ^2^	*P*-value
**Genotype**	**1**	**13.09**	**<0.001**
Sex	**1**	3.46	0.06
Feed restriction	1	1.89	0.17
**Genotype:sex**	**1**	**15.44**	**<<0.001**

Statistics and *P*-values for the explanatory variables correspond to likelihood ratio test (Wald chi-square test) derived from model including all main effects and significant interactions selected on the basis of statistics from the full model in which all predictors and their interactions were included simultaneously. Statistics for the full model is provided in Supplementary Table S3. Significant effects (*α* = 0.05) are in bold.

Model diagnostics showed that fixed effects explained 39.8% of the variability, while the random effect of poultry litter explained only 3.5% of the variability in the data.

### FAs detected in chicken preen gland secretions and factors affecting their relative proportions

A total of 34 FAs were detected in the preen secretions of Ross 308 and ISA Dual genotypes, of which 17 were SFAs, 7 were MUFAs, and 10 were PUFAs (see Supplementary Table S1 for a complete list of FAs and their relative proportions). In addition, of the 34 FAs, we detected 14 FAs that were not reported in previous studies on poultry preen secretions (see Supplementary Table S1).

**Figure 2.**
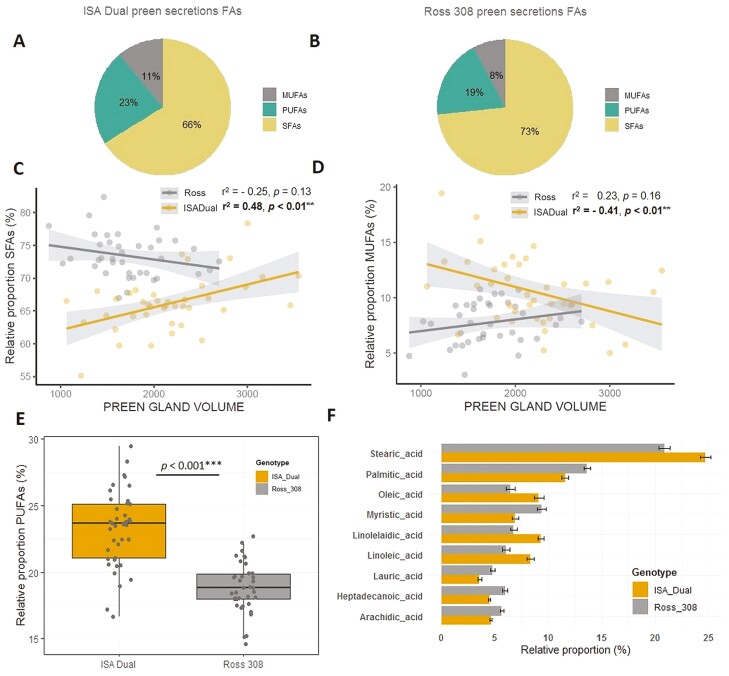
Differences in the relative proportions of FAs detected in the chicken preen gland secretions. Pie charts showing the relative proportion (%) of FA groups found in the Ross 308 (A) and ISA Dual (B) preen gland secretions. The interactive relationship between genotype and preen gland volume on relative proportion of SFAs (C) and MUFAs (D) in preen gland secretions. Box plots showing significant effect of chicken genotype on the relative proportions (%) of PUFAs (E) in preen gland secretions. Significant differences are based on Tukey post hoc test. (F) Bar plot with error bars (mean ± SE) showing differences in the relative proportions (%) of nine most dominant FAs in preen secretions of Ross 308 and ISA Dual chickens.

FAs categories in preen secretions were dominated by SFAs (Ross 308: 73.37%; ISA Dual: 66.13%; Figure 2A and B), followed by PUFAs (Ross 308: 18.86%; ISA Dual: 23.25%; Figure 2A and B) and MUFAs, which accounted for the smallest relative proportion of the total FA profile (Ross 308: 7.76%; ISA Dual: 10.62%; Figure 2A and B).

Of the individual FAs, stearic, palmitic, linoelaidic, oleic, linoleic, myristic, heptadecanoic, arachidic, and lauric acids were the dominant FAs in the preen secretions, with relative proportions ranging from 5% to 25% and varying significantly between Ross 308 and ISA Dual genotypes (Figure 2F; Supplementary Table S1).

Among the tested factors influencing the relative proportion of SFAs and MUFAs in the chicken preen secretions, there was a significant effect of genotype (*P* << 0.001 and *P* < 0.001 for SFAs and MUFAs; Table 2) and the two-way interaction between genotype and preen gland volume (*P* < 0.01 and *P* = 0.015 for SFAs and MUFAs; Table 2). The relative proportion of SFAs was shown to increase with preen gland volume in chickens of the ISA Dual genotype (Pearson’s correlation: *t* = 3.391, *r* = 0.482, *P* = 0.002; Figure 2C), whereas this correlation was nonsignificant in chickens of the Ross 308 genotype (Pearson’s correlation: *t* = −1.542, *r* = −0.248, *P* = 0.131; Figure 2C). On the contrary, the relative proportion of MUFAs was shown to decrease with preen gland volume (Pearson’s correlation: *t* = −2.737, *r* = −0.405, *P* = 0.009; Figure 2D) but again only in chickens of the ISA Dual genotype, whereas this correlation was nonsignificant in chickens of the Ross 308 genotype (Pearson’s correlation: *t* = 1.426, *r* = 0.231, *P* = 0.162; Figure 2D). Feed restriction, sex, and total body weight had no effect on the relative proportion of chicken preen gland secretions SFAs and MUFAs (Table 2).

**Table 2.** LMMs results evaluating the effect of genotype, feed restriction, sex, preen gland volume, total body weight, and their two- and three-way interactions controlled for random effect of experimental pen on the relative proportion of (A) SFAs, (B) MUFAs, and (C) PUFAs in chicken preen gland secretions (*N* = 78)

Explanatory variable	df	*χ* ^2^	*P*-value
A) Saturated fatty acids (SFAs)			
** Genotype**	1	**47.17**	**<<0.001**
** **Feed restriction	1	0.33	0.568
** **Sex	1	0.06	0.806
** **Preen gland volume	1	2.59	0.108
** **Total body weight	1	0.07	0.794
** Genotype:preen gland volume**	1	**8.20**	**<0.01**
B) Monounsaturated fatty acids (MUSFAs)			
** Genotype**	1	**12.12**	**<0.001**
** **Feed restriction	1	0.03	0.858
** **Sex	1	0.99	0.320
** **Preen gland volume	1	2.60	0.107
** **Total body weight	1	1.22	0.270
** Genotype:preen gland volume**	1	**5.98**	**0.015**
C) Polyunsaturated fatty acids (PUFAs)			
** Genotype**	1	**40.54**	**<< 0.001**
** **Feed restriction	1	0.97	0.325
** **Sex	1	1.13	0.288
** **Preen gland volume	1	0.46	0.499
** **Total body weight	1	0.36	0.547

Statistics and *P*-values for the explanatory variables correspond to likelihood ratio tests (Wald chi-square tests) derived from models including all main effects and significant interactions selected on the basis of statistics from the full models in which all predictors were included simultaneously. Statistics for the full models is provided in Supplementary Tables S4A–C. Significant effects (*α* ≥ 0.05) are in bold.

Of all the factors tested affecting relative proportions of PUFAs, only the significant effect of genotype has been found (*P* << 0.001; Table 2) with individuals of the ISA Dual genotype showing a significantly higher relative proportion of PUFAs in preen secretions compared to individuals of the Ross 308 genotype (Tukey post hoc test: *P* < 0.001; Figure 2E). Feed restriction, sex, preen gland volume, or body weight had no effect of on the relative proportions of PUFAs in the preen secretions of ISA Dual and Ross 308 chickens (Table 2).

While fixed effects explained 56.1%, 33.4%, and 46.4% of the variability in the data for the relative proportions of SFA, MUFA, and PUFA in chicken preen gland secretions, respectively, the random effect of littered poultry pen did not affect the explained variability for any of the FA categories.

### VOCs detected in chicken preen gland secretions

**Figure 3.**
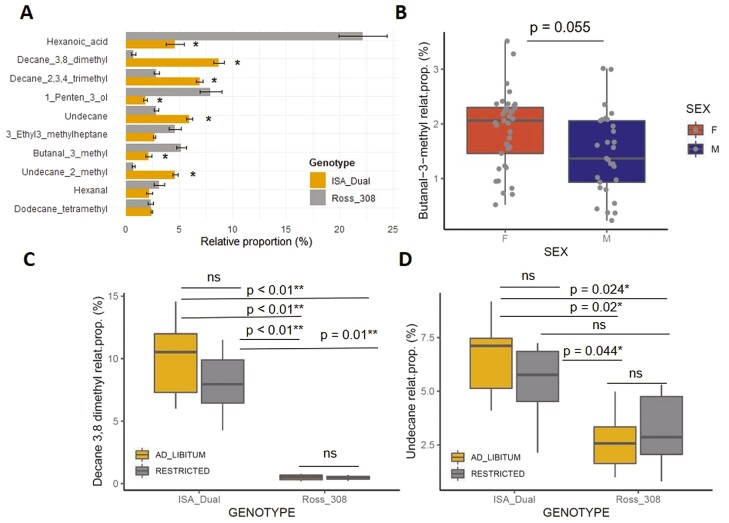
Differences in the relative proportions of the 10 most dominant VOCs detected in the chicken preen gland secretions. (A) Bar plot with error bars (mean ± SE) showing differences in the relative proportions (%) of the 10 most dominant VOCs in preen secretions of Ross 308 and ISA Dual genotypes. VOCs with significant effect of genotype are indicated with asterisk. (B) Box plot showing significant effect of sex on the relative proportions (%) of butanal-3-methyl in both chicken genotypes. Box plots showing significant interactive effect of feed restriction and genotype on the difference in the relative proportion (%) of decane-3,8-dimethyl (C) and undecane (D) in chicken preen secretions. Significant differences are based on Tukey post hoc test.

A total of 77 VOCs were detected in preen secretions of Ross 308 and ISA Dual chickens (see Supplementary Table S2 for a complete list of VOCs and their relative proportions). Although all VOCs have been previously identified, it should be noted that 10 of these 77 VOCs we considered as potential laboratory contaminants (see Supplementary Table S2). Of the remaining 67 VOCs, hexanoic acid was the most dominant, followed by various hydrocarbons (see Supplementary Table S2 and Figure 3A for the 10 most dominant VOCs, whose mean relative proportions ranged from 2.4% to 12%.

### Factors affecting relative proportions of the most dominant VOCs in chicken preen gland secretions

Analysis of factors affecting the relative proportion of the 10 most dominant VOCs in chicken preen secretions revealed a significant effect of genotype, sex, and interaction of genotype and feed restriction, while, as in the case of FAs, feed restriction alone had no effect on the synthesis of the 10 most dominant VOCs in the chicken preen gland (Table 3). For 7 of the 10 most dominant VOCs, their relative proportion was influenced by genotype and differed significantly between ISA Dual and Ross 308 (Table 3 and Figure 3A), while a significant effect of genotype and sex was observed for the relative proportion of butanal-3-methyl (Table 3; Figure 3A and B). For the relative proportions of decane-3,8-dimethyl and undecane, in addition to the effect of genotype, there was also a significant effect of feed restriction, but in interaction with genotype (Table 3; Figure 3C and D), where the relative proportions of decane-3,8-dimethyl and undecane were higher in ISA Dual ad libitum and feed restriction group of chickens compared to Ross 308 ad libitum and feed restriction groups of chickens (Figure 3C and D).

**Table 3.** LMMs results evaluating the effect of genotype, sex, feed restriction, and their two-way interactions controlled for random effect of experimental pen on the relative proportion of the 10 most dominant VOCs in chicken preen gland secretions (*N* = 66)

Explanatory variable		Genotype			Sex			Feed restriction	
**VOC**	df	*χ* ^2^	*P*-value	df	*χ* ^2^	*P*-value	df	*χ* ^2^	*P*-value
Hexanoic acid	1	70.807	**<<0.001**	1	0.600	0.438	1	0.018	0.895
Decane, 3,8-dimethyl-^1^	1	206.657	**<<0.001**	1	0.015	0.903	1	0.120	0.729
Decane, 2,3,4-trimethyl-	1	43.755	**<<0.001**	1	0.033	0.856	1	0.011	0.640
1-penten-3-ol	1	20.599	**<<0.001**	1	0.458	0.499	1	0.089	0.766
Undecane^2^	1	72.162	**<<0.001**	1	0.117	0.733	1	0.365	0.546
3-ethyl-3-methylheptane	1	1.013	0.3142	1	1.660	0.198	1	0.363	0.547
Butanal, 3-methyl-	1	36.486	**<<0.001**	1	7.7675	**<0.01**	1	0.268	0.604
Undecane, 2-methyl-	1	181.162	**<<0.001**	1	0.001	0.975	1	0.637	0.425
Hexanal	1	3.021	0.082	1	2.719	0.099	1	0.710	0.399
2,2,11,11-tetramethyldodecane-	1	0.646	0.422	1	2.125	0.145	1	0.056	0.813

^1^Significant also interaction of genotype:feed restriction: *χ*^2^ = 4.3575; *P-value = 0.037.

^2^Significant also interaction of genotype:feed restriction: *χ*^2^ = 4.3457; *P-value = 0.037.

Statistics and *P*-values for the explanatory variables correspond to likelihood ratio tests (Wald chi-square tests) derived from models including all main effects and significant interactions selected on the basis of statistics from the full models in which all predictors were included simultaneously. Statistics for the full models is provided in Supplementary Tables S5A-I. Significant effects (*α* ≥ 0.05) are in bold.

## Discussion

In our study, we demonstrated an interactive effect of sex and genotype on the size (i.e., volume) of the chicken preen gland, with the preen gland being larger in roosters compared to hens, but only in the ISA Dual genotype. Previous studies on wild and captive birds, including a comparative study on 132 European birds, have shown that the size of preen gland does not differ between the sexes, but increases from the non-breeding to the breeding season in both sexes (Vincze et al., 2013; Ruiz-Rodriguez et al., 2015; Goluke and Caspers, 2017). On the other side, differences in preen gland size between sexes may be species-specific, with larger preen glands observed in females (Martin-Vivaldi et al., 2009; Pap et al., 2010) or males (Moller et al., 2009). Because preen gland size correlates with its secretory potential (Pap et al., 2010), sex differences in preen gland size are mostly a function of the preen gland secretory requirements of a given sex during the reproductive period. Thus, the documented larger preen glands in males of some species are likely related to a greater need for the maintenance of ornamental plumage to increase its signaling potential (Ruiz-Rodriguez et al., 2015; Moreno-Rueda, 2016; Moller and Mateos-Gonzalez, 2019) and larger preen glands in breeding females to their greater ability to provide antimicrobial protection to eggs by coating them with preen oils (Pap et al., 2010; Martin-Vivaldi et al., 2014). However, because there are no studies examining sex differences in the size of the chicken preen gland, we cannot discuss what factors may have played a role in our study showing sex differences in preen gland volume in ISA Dual chickens. Therefore, we hypothesize that preen gland function in the slow-growing dual-purpose ISA Dual genotype is likely to be less affected by intensive genetic selection compared to the fast-growing Ross 308 meat-type genotype.

It is generally assumed that preening itself (Goldstein, 1988) and the synthesis of predominantly waxy compounds into preen secretions are energy-demanding processes (Sweeney et al., 2004; Kolattukudy, 2015) and therefore only individuals in good physical condition may fully invest in the production of preen secretions. This has been supported by studies that have found reduced preen gland volume in ­immunocompetent individuals (Piault et al., 2008; Moreno-Rueda, 2015), individuals with coccidian and malaria infection (Pap et al., 2013; Magallanes et al., 2016), higher microbial loads in plumage (Jacob et al., 2014; Fulop et al., 2016) or having poor body condition (Moreno-Rueda, 2010, 2015, 2016). In our study, only the slow-growing dual-purpose ISA Dual genotype showed sex differences in preen gland volume, while the fast-growing Ross 308 genotype did not. Breeding for selected performance traits in chickens can lead to negative changes in other physiological and morphological parameters, which has been demonstrated particularly in fast-growing meat-type genotypes (Tona et al., 2010; Lambertz et al., 2018; Ben-Gigi et al., 2021). Thus, we hypothesize that chickens of the meat genotype Ross 308 may be in poorer physical condition due to genetic selection for fast growth and rapid feed conversion, and thus are limited in their opportunities to invest in immunity and growth of other morphological structures, such as the preen gland, compared with the ISA Dual genotype. In addition, we found a significantly higher proportion of PUFAs and a genotype-related positive correlation between preen gland volume and the proportion of SFAs in the preen secretion of dual-purpose ISA Dual chickens compared to meat type Ross 308 chickens where this relationship was not significant. Again, we hypothesize that, as in the case of preen gland volume, the differences in the relative proportion of all FAs categories between the Ross 308 and ISA Dual genotypes are most likely due to their long-term selection. As the preen gland and its secretions are not considered in the selection programs, due to the pleiotropy of the genes and the various interactions between them (Wright et al., 2010; Johnsson et al., 2015; Tarsani et al., 2021), changes may also occur in this trait.Moreover, given the energy demands for wax synthesis in preen secretions, the lower relative proportion of FAs in preen secretions that we demonstrated in the Ross 308 genotype would be expected given the selection for rapid growth that likely resulted in suppression of function and synthesis of biochemically active substances in the gland. This assumption is supported by experimental studies on Japanese quail and the Malaysian chicken hybrid Akar Putra, where partial removal of the preen gland resulted in improved growth performance, higher body weight, and lower feed conversion (Jawad et al., 2016a, 2016b; Naji et al., 2020).

In our study, feed restriction had no effect on the relative abundance of any of the fatty acid groups. Feed restriction had genotype-dependent effect on the relative proportion of only two of the ten most dominant VOCs in chicken preen secretions. While the use of different fats and oils in the diet leads to changes in the fat composition of the chicken body (Ajuyah et al., 1991), the fatty acid representation in preen secretions does not reflect the fatty acid levels in the blood and meat (Kanakri et al., 2018). This is evidenced by a study in which geese were fed Sudan III red azo dye for several months, and while the body fat of geese was stained and turned orange, the coloration of uropygial secretions remained unchanged (Pan et al., 1979). Similarly, (Kanakri et al., 2018) found a relationship between diet and the composition of uropygial secretions for only a negligible number of fatty acids tested. Thus, our results are consistent with these previous studies in confirming that diet has little or no effect on the composition of preen secretions in chickens.

In total, we found 34 FAs in the preen secretions of ISA Dual and Ross 308 genotypes, 14 of which have not been detected in the currently available studies on the biochemistry of chicken preen secretions (Apandi and Edwards, 1964; Saito and Gamo, 1968; Pan et al., 1979; Kanakri et al., 2018). These results corroborate with previous studies documenting that preen gland secretions are the site of unique lipids not found elsewhere in the body (Lillard and Toledo, 1976) and that the occurrence of short-chain, branched-chain, or odd-chain fatty acids is typical of preen gland lipids, whereas their occurrence elsewhere is rather sporadic (Saito and Gamo, 1968). Preen secretions of Ross 308 and ISA Dual chickens were dominated by stearic, palmitic, and myristic acid, which is also consistent with previous studies where similar relative proportions of stearic (22.4% to 34.9%) and palmitic (9.9% to 23.2%) acids were found (Sandilands et al., 2004). Only in the study by Kanakri et al. (2018) was the myristic acid the most dominant FA in broiler chicken preen secretions, followed by stearic and palmitic acids. In our study, myristic acid was the third most dominant, thus there is not much discrepancy between our and the findings of this study. Furthermore, we detected a total of 77 VOCs in the preen secretions of ISA Dual and Ross 308 genotype, of which hexanoic acid was the most dominant, followed by various hydrocarbons, alcohol 1-penten-3-ol, and aldehyde butanal, 3-methyl.

For many of the FAs and VOCs we have detected, we can consider their essential role in the welfare of commercially breeding chickens. In particular, hexanoic acid, whose relative proportion was particularly dominant in the secretion of the Ross 308 genotype (22.17%), is a major attractant for poultry red mite (**PRM**; *Dermanyssus gallinae*, De Geer, 1778; Gay et al., 2020). PRM individuals feed on the blood of hens/chickens at night and remain hidden in crevices and cracks during the day. Because PRM parasitism has been found to cause skin irritation, anemia, vascular problems, and even host death (Chauve, 1998; Kilpinen et al., 2005), we assume that Ross 308 individuals whose plumage has been treated with preen secretions dominated by this PRM attractant should potentially suffer more from PRM parasitism. In addition, some of the FAs identified in our study, such as dodecanoic (lauric), decanoic (capric), and butanoic (butyric) acids, have also been demonstrated and patented for their role as aggregation pheromones for PRM (Birkett et al., 2010; Gay et al., 2020). The relative proportion of these FAs in preen secretions also differed between genotypes in our study, with higher values observed for the meat-type genotype Ross 308. Moreover, capric and butyric acids were detected in the preen secretions of chickens for the first time in our study. Their presence in the preen secretions of commercially bred chickens, even at very low concentrations, can act as potent attractants for PRM and other ectoparasites (Birkett et al., 2010; Gay et al., 2020), which may pose problems in welfare and housing management. Contrariwise, aldehydes such as hexanal, decanal, and octanal were documented to act against ectoparasites in free-living birds (Douglas et al., 2001, 2004). As relative proportion of these VOCs was higher in ISA Dual chickens compared to Ross 308, this might also point out better protection against ectoparasite in dual-purpose ISA Dual genotype.

In addition, most of the VOCs contained in preen secretions are important semiochemicals (Soini et al., 2007, 2013; Campagna et al., 2012) that have been shown to play a critical role in odor-mediated intraspecific communication in free-living birds (Whittaker et al., 2010, 2011, 2013). However, this function has also been documented in chickens, where males preferred hens with a preen gland compared to hens without a gland (Hirao et al., 2009). Similarly, female budgerigars (*Melopsittacus undulatus*) used odor signals mediated by the preen secretions to recognize males in a Y-maze experiment (Zhang et al., 2009, 2010). Furthermore, differences in the FAs profile in preen secretions between chickens with pecked and un-pecked feathers likely affected feather odor and taste-making individuals more susceptible to pecking by conspecifics (Sandilands et al., 2004). Thus, based on this evidence, it is highly probable that intraspecific communication mediated by preen secretions may also play a very important role in both poultry and captive parrots. In our study, we did not test antimicrobial potential of chicken preen secretions, yet it is also highly probable that differences in FAs profile between two chicken genotypes may impair antimicrobial potential of preen secretions and increase susceptibility of feathers to degradation by feather-degrading bacteria and other feather or skin disorders. This assumption however needs to be further tested.

Finally, we identified 10 of the 77 VOCs from chicken preen secretions as potential laboratory contaminants. However, we cannot be sure of their origin because some of them, such as nonane, decane, benzene, and benzene-derived hydrocarbons, have been found in the preen secretions of domestic and wild birds (Williams et al., 2003; Bonadonna et al., 2007; Bernier et al., 2008; Gabirot et al., 2018). Since preen secretions can be a reservoir of various environmental pollutants (Lopez-Perea and Mateo, 2019), there is also the possibility that these contaminants we have identified originated in the environment where the experimental chickens were housed.

In conclusion, our study is the first to highlight differences in the chemical composition of preen gland secretions between genetically different chicken genotypes, which may negatively affect various aspects of the welfare of commercially reared chickens. As the implications of this different chemical composition of preen secretions between genetically distinct chickens can only be hypothesized based on previous studies in wild birds, future research should be devoted to both describing differences in the chemical composition of preen secretions in other chicken genotypes, as well as experimentally verifying the effect of the different FA and VOC profiles of preen secretions on the rate of ectoparasite infestation, feather wear quality or direct effect on aggressive behavior and welfare of commercially reared poultry.

## Supplementary Material

skac411_suppl_Supplementary_Data

skac411_suppl_Supplementary_Figure_S1

skac411_suppl_Supplementary_Table_S1

skac411_suppl_Supplementary_Table_S2

skac411_suppl_Supplementary_Table_S3

## Data Availability

The dataset generated and analysed in this study will be available online on Figshare https://figshare.com/ repository under this doi: 10.6084/m9.figshare.21646703.

## Abbreviations

DVB/CAR/PDMS: divinylbenzene/carboxen/polydimethylsiloxane
FA: fatty acid
FID: flame ionization detector
HS: headspace
HS-SPME-MDGC/MS: headspace solid phase micro-extraction multidimensional gas chromatography/mass spectrometry
IS: internal standard
LMM: linear mixed-effects model
MDGC/MS: multidimensional gas chromatography/mass spectrometry
MHUSA: Mother Hens’ Uropygial Secretion Analogue
MUFA: monounsaturated fatty acid
PRM: poultry red mite
PUFA: polysaturated fatty acid
RI: retention indices
SFA: saturated fatty acid
SPME: solid phase microextraction
VOC: volatile organic compound
