# Supplementary material for: Effects of genotype, sex, and feed restriction on the biochemical composition of chicken preen gland secretions and their implications for commercial poultry production
Source: J Anim Sci. 2022 Dec 22;101:skac411. doi: 10.1093/jas/skac411 (PMC9923712; doi:10.1093/jas/skac411)
Supplement: skac411_suppl_Supplementary_Data [file skac411_suppl_supplementary_data.docx]

**Effects of genotype, sex and feed restriction on the biochemical composition of chicken preen gland secretions and their implications for commercial poultry production**

Veronika Gvoždíková Javůrková ^1, 2 *^, Petr Doležal ^1, 3^, Adéla Fraňková ^4^, Monika Horák ^1^, Darina Chodová ^1^, Eva Tůmová ^1^

**Supplementary Method S1**

**Analysis of volatile organic compounds (VOCs)**

*Pre-expeirment testing different SPME fibres, extraction temperatures and equilibration times*

A single sample of the preen gland secretion was thawed, vortexed, separated for identical number of samples and used to test a set of four extraction temperatures (30 °C, 40 °C, 45 °C and 60 °C), four equilibration times (35, 40, 50, 60 min) and four different SPME fibres. The following fibres were tested for maximum VOC extraction - (i) 50 μm divinylbenzene/carboxene/polydimethylsiloxane (DVB/CAR/PDMS) (Supelco, Bellefonte, PA, USA), (ii) 85 μm carboxene (CAR/PDMS), (iii) 65 μm PDMS/divinylbenzene (PDMS/DVB), and (iv) polar 85 μm polyacrylate (PA) from Supelco (Bellefonte, PA, USA). The analytical variability of the VOC extraction and GC-MS analysis was assessed by calculating the variability of total elemental intensities among the 3 replicate samples (data not shown). Optimal extraction parameters for the analysis of non-target VOCs were found for the DVB/CAR/PDMS fibre, and therefore this SPME fibre was used for the analysis of VOCs from chicken preen gland secretions in our study.
