# Supplementary material for: Effects of genotype, sex, and feed restriction on the biochemical composition of chicken preen gland secretions and their implications for commercial poultry production
Source: J Anim Sci. 2022 Dec 22;101:skac411. doi: 10.1093/jas/skac411 (PMC9923712; doi:10.1093/jas/skac411)

**Figure S1**. Diagnostic plots expressing normality of residuals, linearity and posterior predictive check of the LMMs presented in the manuscript as the most robust models using r package *performance*. LMMs evaluating effect of selected explanatory variables and their interactions on **A)** the chicken preen gland volume, **B)** the relative proportion of SFAs**, C)** relative proportion of MUFAs, **D)** relative proportion of PUFAs and particular 10 VOCs **E)**.

**A)**


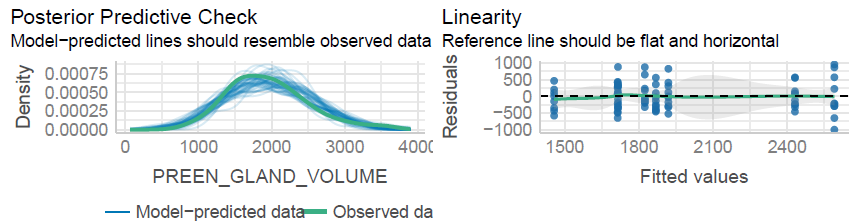


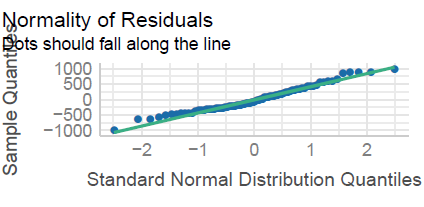


**B)**


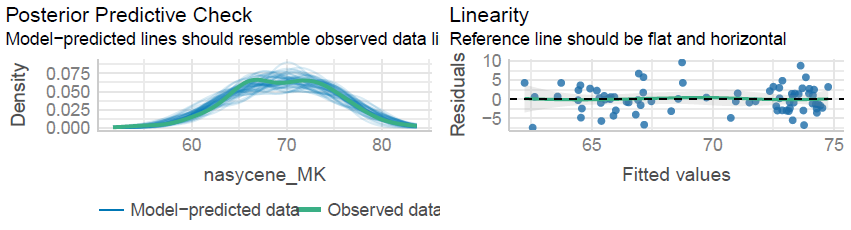


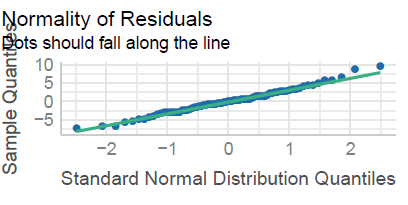


**C)**


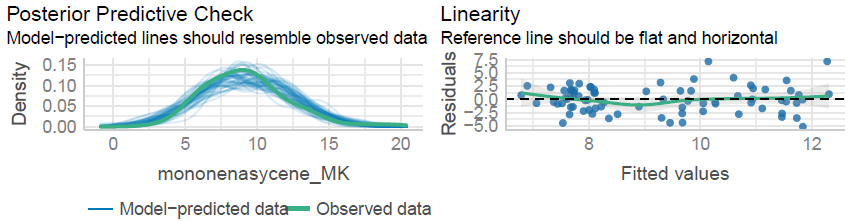


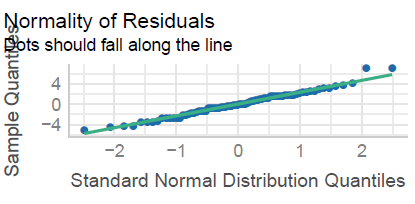


**D)**


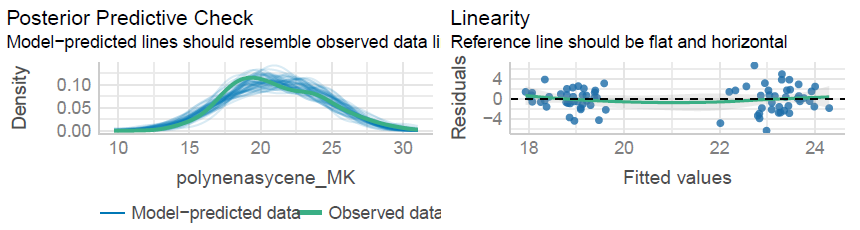


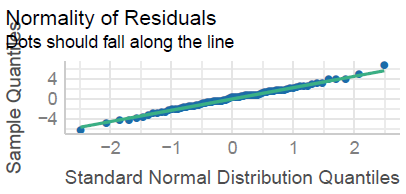


**E)**


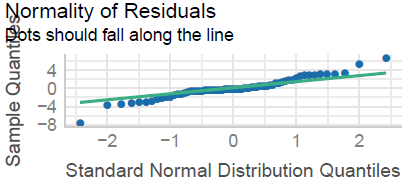

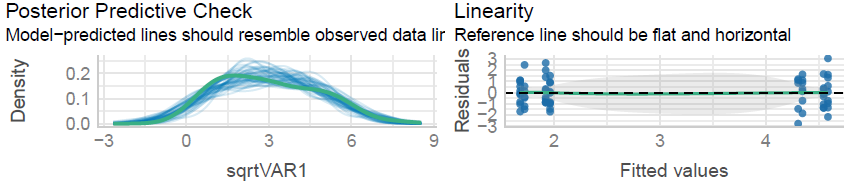


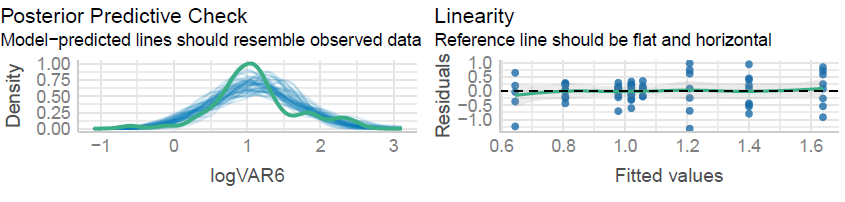

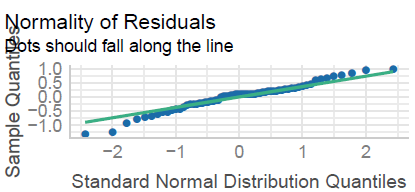

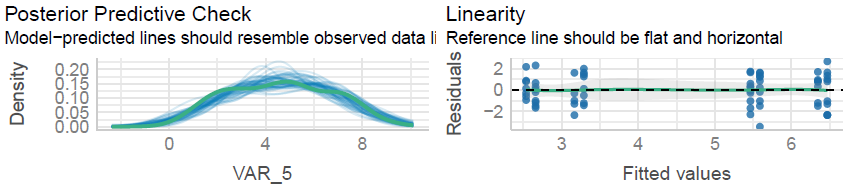

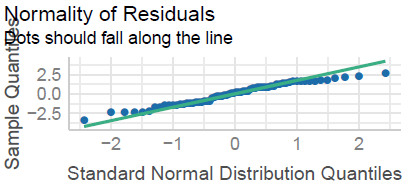

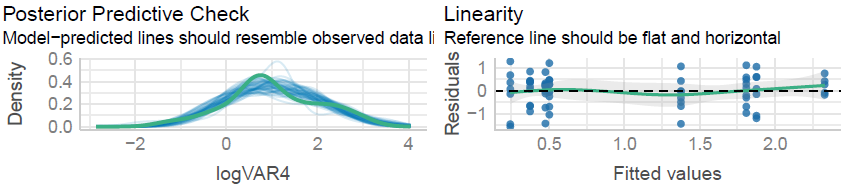

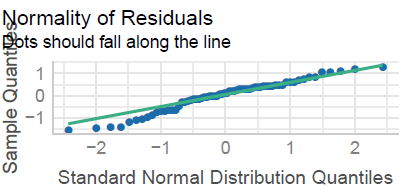

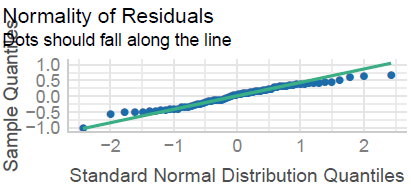

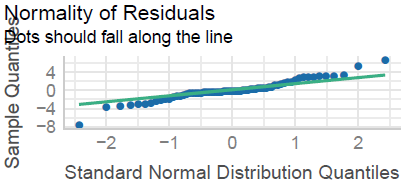


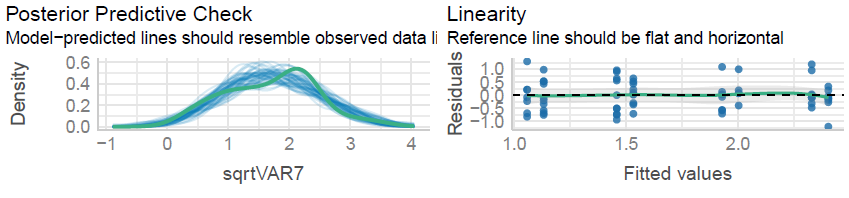

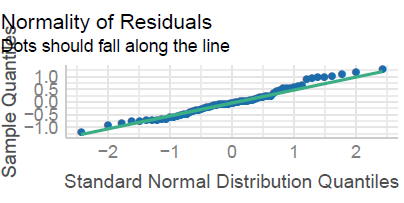

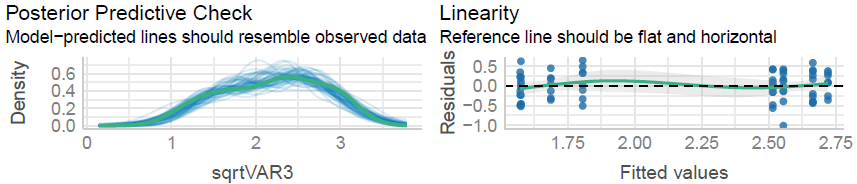


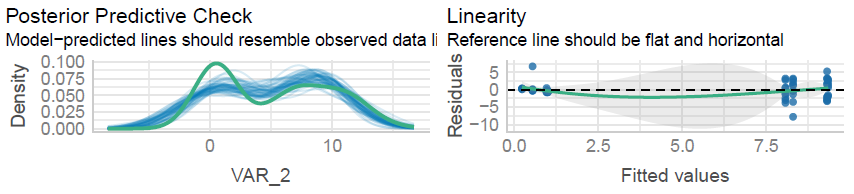


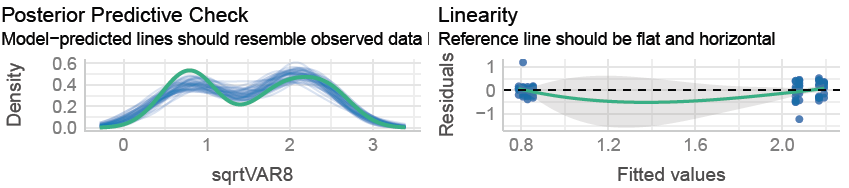

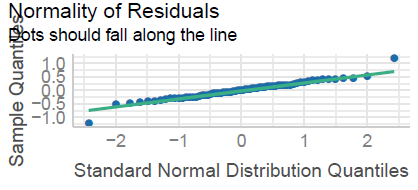


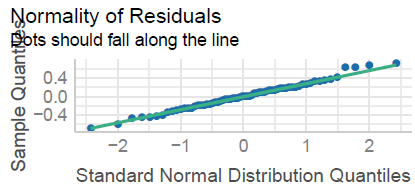

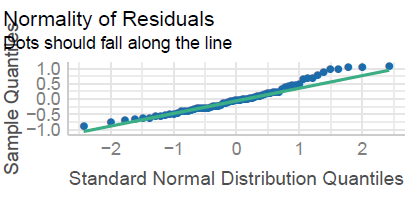

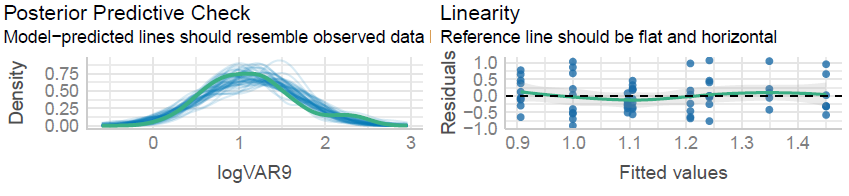


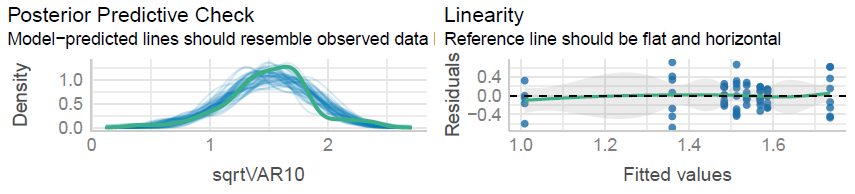

Supplement: skac411_suppl_Supplementary_Figure_S1 [file skac411_suppl_supplementary_figure_s1.docx]
