# Supplementary material for: Effects of genotype, sex, and feed restriction on the biochemical composition of chicken preen gland secretions and their implications for commercial poultry production
Source: J Anim Sci. 2022 Dec 22;101:skac411. doi: 10.1093/jas/skac411 (PMC9923712; doi:10.1093/jas/skac411)
Supplement: skac411_suppl_Supplementary_Table_S1 [file skac411_suppl_supplementary_table_s1.docx]

**Table S1.** List and relative proportions (%) of individual fatty acids (FAs) detected in preen gland secretion of Ross 308 and ISA Dual chickens. FAs not detected in previous studies of chicken preen gland secretions are indicated in bold and with an asterisk.

| **FATTY ACID NAME** | **CHICKEN GENOTYPE** |  |
| --- | --- | --- |
|  |  |  |
| **Saturated FAs (SFAs)** | **Ross 308** (relative proportion %) | **ISA Dual** (relative proportion %) |
|  |  |  |
| stearic acid | 20.91 | 24.77 |
| palmitic acid | 13.66 | 11.6 |
| myristic acid | 9.39 | 6.94 |
| heptadecanoic acid | 5.97 | 4.53 |
| arachidic acid | 5.68 | 4.67 |
| lauric acid | 4.83 | 3.59 |
| **capric acid*** | 3.04 | 1.74 |
| **caproic (hexanoic) acid*** | 1.99 | 1.09 |
| behenic acid | 1.85 | 1.64 |
| tricosylic acid | 1.62 | 2.27 |
| **butyric acid*** | 1.08 | 0.53 |
| pentadecylic acid | 1.03 | 0.79 |
| tridecylic acid | 0.89 | 0.56 |
| undecanoic acid | 0.67 | 0.38 |
| **caprylic acid*** | 0.45 | 0.25 |
| lignoceric acid | 0.21 | 0.29 |
| heneicosylic acid | 0.1 | 0.49 |
|  |  |  |
| **Polyunsaturated FAs (PUFAs)** | **Ross 308** (relative proportion % ) | **ISA Dual** (relative proportion %) |
|  |  |  |
| **linolelaidic acid*** | 6.79 | 9.31 |
| linoleic acid | 6.07 | 8.37 |
| **cis-eicosa-11,14-dienoic acid*** | 1.53 | 1.41 |
| **cis-eicosa-11,14,17-trienoic acid*** | 1.29 | 0.98 |
| **cis-docosa-4,7,10,13,16,19-hexaenoic acid*** | 0.93 | 0.7 |
| **α-Linolenic acid*** | 0.7 | 0.96 |
| **γ-linolenic acid*** | 0.61 | 0.39 |
| **cis-eicosa-8,11,14-trienoic acid*** | 0.54 | 0.62 |
| **cis-docosa-13,16-dienoic + cis-eicosa-5,8,11,14,17-pentaenoic acid*** | 0.4 | 0.49 |
| **arachidonic acid*** | 0 | 0.01 |
|  |  |  |
| **Monounsaturated FAs (MUFAs)** | **Ross 308** (relative proportion % ) | **ISA Dual** (relative proportion %) |
|  |  |  |
| oleic acid | 6.47 | 9.15 |
| palmitoleic acid | 0.57 | 0.89 |
| **erucic acid*** | 0.51 | 0.29 |
| **nervonic acid*** | 0.2 | 0.16 |
| **elaidic acid*** | 0.01 | 0.06 |
| myristoleic acid | 0 | 0.02 |
| **cis-10-pentadeceonic acid*** | 0 | 0.04 |
|  |  |  |
