# Supplementary material for: Effects of genotype, sex, and feed restriction on the biochemical composition of chicken preen gland secretions and their implications for commercial poultry production
Source: J Anim Sci. 2022 Dec 22;101:skac411. doi: 10.1093/jas/skac411 (PMC9923712; doi:10.1093/jas/skac411)
Supplement: skac411_suppl_Supplementary_Table_S2 [file skac411_suppl_supplementary_table_s2.docx]

**Table S2.** List and relative proportions (%) of individual volatile organic compounds (VOCs) detected in preen gland secretion of Ross 308 and ISA Dual chicken genotypes. The retention indices I_x_ (comp.) were calculated according to the definition of Van den Dool and Kratz for a non-isothermal analysis. I_x_ (lit.) means retention indices taken from literature. References from the NIST Standard Reference Database 1A v14 are in brackets. Df means defined retention indices for n-alkanes. Rt (min) are the retention time of compounds. VOCs considered as potential laboratory contaminants are indicated in bold and with an asterisk.

| **Order** | **Chemical compound (IUPAC)** | **Rt (min.)** | **l_x_ (comp.)** | **I_x_ (lit.)** | **Mean relat. prop. (%)** | **ISA Dual rel.prop.(%)** | **Ross308 rel.prop.(%)** | **Type of compound** |
| --- | --- | --- | --- | --- | --- | --- | --- | --- |
| 1 | hexanoic acid | 21.295 | 977 | 977 (1) | 12.46 | 4.60 | 22.17 | carboxylic acid |
| 2 | **hexane, 2,2,5-trimethyl- *** | 23.375 | 799 | 789 (2) | 6.77 | 11.40 | 1.08 | hydrocarbon |
| 3 | **n-hexane*** | 10.317 | 598 | 600 (df) | 6.32 | 11.17 | 0.34 | hydrocarbon |
| 4 | decane, 3,8-dimethyl- | 24.497 | 1121 | 1133 (3) | 5.13 | 8.70 | 0.72 | hydrocarbon |
| 5 | decane, 2,3,4-trimethyl- | 24.16 | 1205 | 1260 (4) | 5.11 | 6.92 | 2.87 | hydrocarbon |
| 6 | 1-penten-3-ol | 13.035 | 685 | 686 (5) | 4.58 | 1.84 | 7.95 | alcohol |
| 7 | undecane | 24.053 | 1100 | 1100 (df) | 4.55 | 5.94 | 2.84 | hydrocarbon |
| 8 | **benzene, 1,3-bis(1,1-dimethylethyl)-*** | 27.647 | 1276 | 1245(6) | 3.58 | 3.89 | 3.20 | hydrocarbon |
| 9 | 3-ethyl-3-methylheptane | 23.243 | 1063 | n/a | 3.55 | 2.69 | 4.61 | hydrocarbon |
| 10 | butanal, 3-methyl- | 12.185 | 658 | 658 (7) | 3.5 | 2.12 | 5.20 | aldehyde |
| 11 | **benzene*** | 12.575 | 670 | 663 (8) | 3.07 | 2.58 | 3.67 | hydrocarbon |
| 12 | undecane, 2-methyl- | 24.653 | 1159 | 1163 (4) | 2.89 | 4.64 | 0.74 | hydrocarbon |
| 13 | hexanal | 16.712 | 803 | 803 (9) | 2.63 | 2.23 | 3.11 | aldehyde |
| 14 | 2,2,11,11-tetramethyldodecane | 22.375 | 1024 | n/a | 2.37 | 2.41 | 2.32 | hydrocarbon |
| 15 | 1-pentanol | 15.648 | 768 | 768 (10) | 1.75 | 1.55 | 1.99 | alcohol |
| 16 | 2,2'-bifuran, octahydro- | 23.91 | 1093 | n/a | 1.53 | 2.64 | 0.16 | furane |
| 17 | 2(3h)-furanone, dihydro-4-methyl- | 20.147 | 930 | 916 (11) | 1.46 | 1.25 | 1.71 | furane |
| 18 | 3-pentanone | 13.42 | 697 | 700 (10) | 1.21 | 0.70 | 1.85 | ketone |
| 19 | heptane, 3,3,4-trimethyl- | 22.358 | 1023 | n/a | 1.2 | 0.62 | 1.93 | hydrocarbon |
| 20 | 3-octanone | 21.587 | 989 | 989 (12) | 1.14 | 1.01 | 1.31 | ketone |
| 21 | benzaldehyde | 21.665 | 992 | 990(13) | 1.13 | 1.04 | 1.23 | aldehyde |
| 22 | linalool | 24.27 | 1110 | 1112 (14) | 1.11 | 1.20 | 1.00 | alcohol |
| 23 | 1-heptanol | 21.183 | 973 | 971(15) | 1.09 | 1.02 | 1.19 | alcohol |
| 24 | butanoic acid, 2-methyl- | 18.038 | 851 | 850 (8) | 1.03 | 0.73 | 1.39 | carboxylic acid |
| 25 | 1-hexanol | 18.6 | 871 | 871(16) | 0.96 | 0.71 | 1.27 | alcohol |
| 26 | pentanal | 13.503 | 700 | 700 (17) | 0.94 | 0.70 | 1.22 | aldehyde |
| 27 | 2-hepten-1-ol, (e)- | 21.033 | 966 | n/a | 0.93 | 0.76 | 1.13 | alcohol |
| 28 | butanoic acid, 3-methyl- | 17.758 | 840 | 839 (8) | 0.9 | 1.25 | 0.46 | carboxylic acid |
| 29 | decane, 2,2-dimethyl- | 22.62 | 1035 | n/a | 0.89 | 1.50 | 0.13 | hydrocarbon |
| 30 | 2-nonanone | 24.003 | 1097 | 1096 (18) | 0.86 | 0.77 | 0.97 | ketone |
| 31 | dodecane | 26.113 | 1200 | 1200 (df) | 0.72 | 0.65 | 0.81 | hydrocarbon |
| 32 | 2-heptanone | 19.212 | 893 | 893 (19) | 0.72 | 0.59 | 0.89 | ketone |
| 33 | heptane, 2,4-dimethyl- | 17.275 | 823 | 820 (20) | 0.7 | 0.17 | 1.36 | hydrocarbon |
| 34 | heptanal | 19.567 | 906 | 906 (19) | 0.69 | 0.63 | 0.77 | aldehyde |
| 35 | **nonane*** | 19.378 | 899 | 900 (df) | 0.65 | 0.60 | 0.72 | hydrocarbon |
| 36 | butanal, 2-methyl- | 12.51 | 668 | 668(21) | 0.56 | 0.35 | 0.83 | aldehyde |
| 37 | 2(3h)-furanone, 5-hexyldihydro- | 30.375 | 1414 | 1414 (22) | 0.51 | 0.33 | 0.72 | furane, ketone |
| 38 | 1-octen-3-ol | 21.442 | 983 | 983 (23) | 0.49 | 0.44 | 0.57 | alcohol |
| 39 | betaine | 6.987 | 492 | n/a | 0.49 | 0.12 | 0.93 | amino acid derivative |
| 40 | nonane, 2-methyl- | 21.028 | 966 | 962(22) | 0.46 | 0.17 | 0.82 | hydrocarbon |
| 41 | pyridine | 15.305 | 758 | 757 (24) | 0.45 | 0.23 | 0.72 | heterocyclic compound |
| 42 | decane, 4-methyl- | 22.028 | 1048 | 1059 (25) | 0.45 | 0.53 | 0.35 | hydrocarbon |
| 43 | undecane, 3,6-dimethyl- | 26.353 | 1212 | 1210 (26) | 0.4 | 0.07 | 0.79 | hydrocarbon |
| 44 | **decane*** | 21.848 | 1000 | 1000 (df) | 0.37 | 0.30 | 0.46 | hydrocarbon |
| 45 | 1-pentanol | 14.685 | 738 | 735 (10) | 0.33 | 0.15 | 0.55 | alcohol |
| 46 | acetaldehyde | 6.6 | 480 | n/a | 0.31 | 0.19 | 0.47 | aldehyde |
| 47 | 2,4-dimethyl-1-heptene | 17.87 | 844 | 836 (27) | 0.3 | 0.31 | 0.30 | hydrocarbon |
| 48 | butanoic acid | 16.21 | 786 | 780 (28) | 0.27 | 0.02 | 0.57 | carboxylic acid |
| 49 | nonane, 5-(1-methylpropyl)- | 25.217 | 1156 | n/a | 0.25 | 0.28 | 0.22 | hydrocarbon |
| 50 | acetoin | 13.973 | 715 | 718 (29) | 0.25 | 0.12 | 0.40 | ketone, alcohol |
| 51 | tetradecane, 5-methyl- | 27.363 | 1462 | 1454 (25) | 0.23 | 0.02 | 0.49 | hydrocarbon |
| 52 | dodecane, 2,6,11-trimethyl- | 28.587 | 1323 | n/a | 0.22 | 0.13 | 0.33 | hydrocarbon |
| 53 | undecanal | 28.6 | 1324 | 1319 (30) | 0.22 | 0.13 | 0.32 | aldehyde |
| 54 | 3-octen-2-one, (e)- | 22.905 | 1048 | 1036 (31) | 0.21 | 0.30 | 0.11 | ketone |
| 55 | propanal | 7.91 | 522 | 506 (32) | 0.21 | 0.17 | 0.27 | aldehyde |
| 56 | dodecane, 4-methyl- | 26.543 | 1241 | 1259 (25) | 0.18 | 0.03 | 0.38 | hydrocarbon |
| 57 | carbonic acid, dimethyl ester | 11.012 | 621 | 620 (33) | 0.16 | 0.07 | 0.26 | ester |
| 58 | **tridecane*** | 28.128 | 1300 | 130 (df) | 0.16 | 0.11 | 0.21 | hydrocarbon |
| 59 | 1-butanol | 12.435 | 666 | 668 (10) | 0.15 | 0.09 | 0.21 | alcohol |
| 60 | 2-penten-1-ol, (z)- | 15.773 | 772 | 767 (10) | 0.14 | 0.11 | 0.18 | alcohol |
| 61 | dodecane, 4,6-dimethyl- | 27.962 | 1291 | n/a | 0.12 | 0.09 | 0.16 | hydrocarbon |
| 62 | butanal | 10.342 | 599 | 598 (34) | 0.12 | 0.11 | 0.12 | aldehyde |
| 63 | propanal, 2-methyl- | 9.315 | 566 | 558 (21) | 0.11 | 0.07 | 0.15 | aldehyde |
| 64 | **toluene*** | 16.008 | 770 | 771 (35) | 0.11 | 0.11 | 0.11 | hydrocarbon |
| 65 | 1-dodecanol | 31.915 | 1492 | 1487 (36) | 0.09 | 0.09 | 0.09 | alcohol |
| 66 | 5-hexenal | 16.368 | 791 | n/a | 0.08 | 0.11 | 0.04 | aldehyde |
| 67 | 1-butanol, 2-methyl- | 14.842 | 743 | 743 (37) | 0.07 | 0.03 | 0.12 | alcohol |
| 68 | thiazole | 15.018 | 748 | 735 (38) | 0.07 | 0.04 | 0.11 | heterocyclic compound |
| 69 | 1-octene | 16.405 | 793 | 789 (25) | 0.07 | 0.07 | 0.07 | hydrocarbon |
| 70 | 2-octanone | 20.442 | 982 | 984 (39) | 0.06 | 0.07 | 0.04 | ketone |
| 71 | 1-undecanol | 29.863 | 1388 | 1387 (36) | 0.05 | 0.04 | 0.07 | alcohol |
| 72 | 1-nonanol | 25.668 | 1178 | 1175 (40) | 0.05 | 0.04 | 0.06 | alcohol |
| 73 | 2-butanone, 3-methyl- | 10.108 | 692 | 673 (41) | 0.04 | 0.05 | 0.04 | ketone |
| 74 | **2-hexene, 4,4,5-trimethyl-*** | 17.517 | 832 | n/a | 0.03 | 0.03 | 0.03 | hydrocarbon |
| 75 | dodecane, 4,6-dimethyl- | 26.783 | 1333 | 1325 (42) | 0.03 | 0.03 | 0.03 | hydrocarbon |
| 76 | 1-pentene, 2-methyl- | 11.403 | 603 | 584(43) | 0.02 | 0.03 | 0.00 | hydrocarbon |
| 77 | **pentane, 3-methyl-*** | 9.867 | 584 | 580 (8) | 0.01 | 0.02 | 0.00 | hydrocarbon |

**References for retention indices**

1. Lalel HJD, Singh Z, Tan SC. Glycosidically-bound aroma volatile compounds in the skin and pulp of ‘Kensington Pride’ mango fruit at different stages of maturity. Postharvest Biology and Technology. 2003;29(2):205-18.

2. Insausti K, Goñi V, Petri E, Gorraiz C, Beriain MJ. Effect of weight at slaughter on the volatile compounds of cooked beef from Spanish cattle breeds. Meat Science. 2005;70(1):83-90.

3. Kotowska U, Żalikowski M, Isidorov VA. HS-SPME/GC–MS analysis of volatile and semi-volatile organic compounds emitted from municipal sewage sludge. Environmental Monitoring and Assessment. 2012;184(5):2893-907.

4. Cardeal ZL, Gomes da Silva MD, Marriott PJ. Comprehensive two-dimensional gas chromatography/mass spectrometric analysis of pepper volatiles. Rapid Commun Mass Spectrom. 2006;20(19):2823-36.

5. Methven L, Tsoukka M, Oruna-Concha MJ, Parker JK, Mottram DS. Influence of Sulfur Amino Acids on the Volatile and Nonvolatile Components of Cooked Salmon (Salmo salar). Journal of Agricultural and Food Chemistry. 2007;55(4):1427-36.

6. Iraqi R, Vermeulen C, Benzekri A, Bouseta A, Collin S. Screening for Key Odorants in Moroccan Green Olives by Gas Chromatography−Olfactometry/Aroma Extract Dilution Analysis. Journal of Agricultural and Food Chemistry. 2005;53(4):1179-84.

7. Dallüge J, van Stee LLP, Xu X, Williams J, Beens J, Vreuls RJJ, et al. Unravelling the composition of very complex samples by comprehensive gas chromatography coupled to time-of-flight mass spectrometry: Cigarette smoke. Journal of Chromatography A. 2002;974(1):169-84.

8. Engel E, Ratel J. Correction of the data generated by mass spectrometry analyses of biological tissues: Application to food authentication. Journal of Chromatography A. 2007;1154(1):331-41.

9. Ruiz Perez-Cacho P, Mahattanatawee K, Smoot JM, Rouseff R. Identification of Sulfur Volatiles in Canned Orange Juices Lacking Orange Flavor. Journal of Agricultural and Food Chemistry. 2007;55(14):5761-7.

10. Pino JA, Mesa J, Muñoz Y, Martí MP, Marbot R. Volatile Components from Mango (Mangifera indica L.) Cultivars. Journal of Agricultural and Food Chemistry. 2005;53(6):2213-23.

11. Lee C-J, DeMilo AB, Moreno DS, Mangan RL. Identification of the Volatile Components of E802 Mazoferm Steepwater, a Condensed Fermented Corn Extractive Highly Attractive to the Mexican Fruit Fly (Diptera:  Tephritidae). Journal of Agricultural and Food Chemistry. 1997;45(6):2327-31.

12. Flamini G, Cioni PL, Morelli I. Composition of the essential oils and in vivo emission of volatiles of four Lamium species from Italy: L. purpureum, L. hybridum, L. bifidum and L. amplexicaule. Food Chemistry. 2005;91(1):63-8.

13. Elmore JS, Erbahadir MA, Mottram DS. Comparison of Dynamic Headspace Concentration on Tenax with Solid Phase Microextraction for the Analysis of Aroma Volatiles. Journal of Agricultural and Food Chemistry. 1997;45(7):2638-41.

14. Loayza I, Abujder D, Aranda R, Jakupovic J, Collin G, Deslauriers H, et al. Essential oils of Baccharis salicifolia, B. latifolia and B. dracunculifolia. Phytochemistry. 1995;38(2):381-9.

15. Wu S, Zorn H, Krings U, Berger RG. Volatiles from submerged and surface-cultured beefsteak fungus, Fistulina hepatica. Flavour and Fragrance Journal. 2007;22(1):53-60.

16. Zhao J, Liu J, Zhang X, Liu Z, Tsering T, Zhong Y, et al. Chemical composition of the volatiles of three wild Bergenia species from western China. Flavour and Fragrance Journal. 2006;21(3):431-4.

17. Moio L, Rillo L, Ledda A, Addeo F. Odorous Constituents of Ovine Milk in Relationship to Diet. Journal of Dairy Science. 1996;79(8):1322-31.

18. Kallio M, Jussila M, Rissanen T, Anttila P, Hartonen K, Reissell A, et al. Comprehensive two-dimensional gas chromatography coupled to time-of-flight mass spectrometry in the identification of organic compounds in atmospheric aerosols from coniferous forest. Journal of Chromatography A. 2006;1125(2):234-43.

19. Flamini G, Tebano M, Cioni PL, Bagci Y, Dural H, Ertugrul K, et al. A multivariate statistical approach to Centaurea classification using essential oil composition data of some species from Turkey. Plant Systematics and Evolution. 2006;261(1):217-28.

20. Wang Z, Fingas M, Li K. Fractionation of a Light Crude Oil and Identification and Quantitation of Aliphatic, Aromatic, and Biomarker Compounds by GC-FID and GC-MS, Part II. Journal of Chromatographic Science. 1994;32(9):367-82.

21. Carrapiso AI, Ventanas J, García C. Characterization of the Most Odor-Active Compounds of Iberian Ham Headspace. Journal of Agricultural and Food Chemistry. 2002;50(7):1996-2000.

22. Sun G, Stremple P. Retention index characterization of flavor, fragrance, and many other compounds on DB-1 and DB-XLB 2003 [Available from: <http://www.chem.agilent.com/cag/cabu/pdf/b-0279.pdf>].

23. Cho IH, Namgung HJ, Choi HK, Kim YS. Volatiles and key odorants in the pileus and stipe of pine-mushroom (Tricholoma matsutake Sing.). Food Chemistry. 2008;106(1):71-6.

24. Oruna-Concha MJ, Duckham SC, Ames JM. Comparison of Volatile Compounds Isolated from the Skin and Flesh of Four Potato Cultivars after Baking. Journal of Agricultural and Food Chemistry. 2001;49(5):2414-21.

25. Zaikin VG, Borisov RS. Chromatographic-mass spectrometric analysis of Fishcer-Tropsch synthesis products. J. Anal. Chem. USSR (Engl. Transl.); 2002. p. 544-51.

26. Ramarathnam N, Rubin LJ, Diosady LL. Studies on meat flavor. 4. Fractionation, characterization, and quantitation of volatiles from uncured and cured beef and chicken. Journal of Agricultural and Food Chemistry. 1993;41(6):939-45.

27. Pang T, Zhu S, Lu X, Xu G. Identification of unknown compounds on the basis of retention index data in comprehensive two-dimensional gas chromatography. Journal of Separation Science. 2007;30(6):868-74.

28. Pino J, Marbot R, Rosado A, Vázquez C. Volatile constituents of fruits of Garcinia dulcis Kurz. from Cuba. Flavour and Fragrance Journal. 2003;18(4):271-4.

29. Lozano PR, Drake M, Benitez D, Cadwallader KR. Instrumental and Sensory Characterization of Heat-Induced Odorants in Aseptically Packaged Soy Milk. Journal of Agricultural and Food Chemistry. 2007;55(8):3018-26.

30. Dickschat JS, Wagner-Döbler I, Schulz S. The Chafer Pheromone Buibuilactone and Ant Pyrazines are also Produced by Marine Bacteria. Journal of Chemical Ecology. 2005;31(4):925-47.

31. Splivallo R, Bossi S, Maffei M, Bonfante P. Discrimination of truffle fruiting body versus mycelial aromas by stir bar sorptive extraction. Phytochemistry. 2007;68(20):2584-98.

32. Engel E, Baty C, le Corre D, Souchon I, Martin N. Flavor-Active Compounds Potentially Implicated in Cooked Cauliflower Acceptance. Journal of Agricultural and Food Chemistry. 2002;50(22):6459-67.

33. Rotsatschakul P, Visesanguan W, Smitinont T, Chaiseri S. *Changes in volatile compounds during fermentation of nham (Thai fermented sausage)*. Int. Food Res. J.; 2009. p. 391-414.

34. Majcher MA, Jeleń HH. Effect of Cysteine and Cystine Addition on Sensory Profile and Potent Odorants of Extruded Potato Snacks. Journal of Agricultural and Food Chemistry. 2007;55(14):5754-60.

35. Bredie WLP, Mottram DS, Guy RCE. Aroma Volatiles Generated during Extrusion Cooking of Maize Flour. Journal of Agricultural and Food Chemistry. 1998;46(4):1479-87.

36. Aaslyng MD, Elmore JS, Mottram DS. Comparison of the Aroma Characteristics of Acid-Hydrolyzed and Enzyme-Hydrolyzed Vegetable Proteins Produced from Soy. Journal of Agricultural and Food Chemistry. 1998;46(12):5225-31.

37. Moio L, Piombino P, Addeo F. Odour-impact compounds of Gorgonzola cheese. Journal of Dairy Research. 2000;67(2):273-85.

38. Parker JK, Hassell GME, Mottram DS, Guy RCE. Sensory and Instrumental Analyses of Volatiles Generated during the Extrusion Cooking of Oat Flours. Journal of Agricultural and Food Chemistry. 2000;48(8):3497-506.

39. Sampaio TS, L. Nogueira PC. Volatile components of mangaba fruit (Hancornia speciosa Gomes) at three stages of maturity. Food Chemistry. 2006;95(4):606-10.

40. Kim TH, Shin JH, Baek HH, Lee HJ. Volatile flavour compounds in suspension culture of Agastache rugosa Kuntze (Korean mint). Journal of the Science of Food and Agriculture. 2001;81(6):569-75.

41. Owens JD, Allagheny N, Kipping G, Ames JM. Formation of Volatile Compounds During Bacillus subtilis Fermentation of Soya Beans. Journal of the Science of Food and Agriculture. 1997;74(1):132-40.

42. Liu Y, Xu X-l, Zhou G-h. Comparative study of volatile compounds in traditional Chinese Nanjing marinated duck by different extraction techniques. International Journal of Food Science & Technology. 2007;42(5):543-50.

43. Xu X, Stee LLP, Williams J, Beens J, Adahchour M, Vreuls RJJ, et al. Comprehensive two-dimensional gas chromatography (GC × GC) measurements of volatile organic compounds in the atmosphere. Atmos Chem Phys. 2003;3(3):665-82.
