# Supplementary material for: Effects of genotype, sex, and feed restriction on the biochemical composition of chicken preen gland secretions and their implications for commercial poultry production
Source: J Anim Sci. 2022 Dec 22;101:skac411. doi: 10.1093/jas/skac411 (PMC9923712; doi:10.1093/jas/skac411)
Supplement: skac411_suppl_Supplementary_Table_S3 [file skac411_suppl_supplementary_table_s3.docx]

**Table S3.** Results of full LMM evaluating the effect of of genotype, feed restriction, sex and their two-way interactions controlled for random effect of experimental pen on the chicken preen gland volume (N=78). Significant effects (α = 0.05) are in bold.

|  |  |  |  |
| --- | --- | --- | --- |
| Explanatory variable | Df | χ^2^ | *P-*value |
| **genotype** | **1** | **6.55** | **0.01** |
| sex | 1 | 1.58 | 0.21 |
| feed restriction | **1** | 0.94 | 0.33 |
| **genotype:sex** | **1** | **7.56** | **<0.01** |
| genotype:feed restriction | 1 | 0.05 | 0.83 |
| sex:feed restriction | 1 | 0.42 | 0.52 |
|  |  |  |  |

**Table S4.** Results of full LMMs evaluating the effect of genotype, feed restriction, sex, preen gland volume, total body weight, and their two- and three-way interactions controlled for random effect of experimental pen on the relative proportion of **(A)** SFAs, **(B)** MUFAs and **(C)** PUFAs in chicken preen gland secretions (N=78). Significant effects (α = 0.05) are in bold.

| **A)** |  |  |  |
| --- | --- | --- | --- |
| **Saturated fatty acids (SFAs)** |  |  |  |
| Explanatory variable | Df | χ2 | *P*-value |
| **genotype** | 1 | **24.98** | **<<0.001** |
| sex | 1 | 0.14 | 0.705 |
| feed restriction | 1 | 0.19 | 0.662 |
| preen gland volume | 1 | 3.67 | 0.055 |
| total body weight | 1 | 1.85 | 0.173 |
| genotype:sex | 1 | 1.88 | 0.170 |
| genotype:feed restriction | 1 | 0.06 | 0.802 |
| **genotype:preen gland volume** | 1 | **6.49** | **0.011** |
| genotype:total body weight | 1 | 0.32 | 0.574 |
| sex:feed restriction | 1 | 0.03 | 0.857 |
| sex:preen gland volume | 1 | 0.06 | 0.801 |
| sex:total body weight | 1 | 0.05 | 0.820 |
| genotype:sex:preen gland volume | 1 | 2.60 | 0.107 |
|  |  |  |  |
| **B)** |  |  |  |
| **Monounsaturated fatty acids (MUSFAs)** |  |  |  |
| Explanatory variable | Df | χ2 | *P*-value |
| **genotype** | 1 | **5.23** | **0.022** |
| sex | 1 | 0.70 | 0.401 |
| feed restriction | 1 | 0.00 | 0.96 |
| preen gland volume | 1 | 3.75 | **0.053** |
| total body weight | 1 | 2.93 | 0.087 |
| genotype:sex | 1 | 0.65 | 0.420 |
| genotype:feed restriction | 1 | 0.02 | 0.893 |
| **genotype:preen gland volume** | 1 | 5.97 | **0.015** |
| genotype:total body weight | 1 | 0.31 | 0.579 |
| sex:feed restriction | 1 | 0.02 | 0.875 |
| sex:preen gland volume | 1 | 0.23 | 0.635 |
| sex:total body weight | 1 | 0.22 | 0.637 |
| genotype:sex:preen gland volume | 1 | 0.96 | 0.327 |
|  |  |  |  |
| **C)** |  |  |  |
| **Polyunsaturated fatty acids (PUSFAs)** |  |  |  |
| Explanatory variable | Df | χ2 | *P*-value |
| **genotype** | 1 | **25.33** | **<< 0.001** |
| sex | 1 | 0.17 | 0.682 |
| feed restriction | 1 | 0.71 | 0.399 |
| preen gland volume | 1 | 0.65 | 0.421 |
| total body weight | 1 | 0.03 | 0.869 |
| genotype:sex | 1 | 1.38 | 0.240 |
| genotype:feed restriction | 1 | 0.31 | 0.581 |
| genotype:preen gland volume | 1 | 1.35 | 0.245 |
| genotype:total body weight | 1 | 0.10 | 0.750 |
| sex:feed restriction | 1 | 0.02 | 0.890 |
| sex:preen gland volume | 1 | 0.75 | 0.388 |
| sex:total body weight | 1 | 0.63 | 0.426 |
| genotype:sex:preen gland volume | 1 | 1.59 | 0.207 |

**Table S5**. Results of full LMMs evaluating the effect of sex, genotype, and feed restriction and their two-way interactions on the relative proportion of the ten most dominant VOCs **(A)-(I)** in chicken preen gland secretions (N=66). Statistics and p-values for the explanatory variables correspond to likelihood ratio tests (Wald chi-square tests). Significant effects (α = 0.05) are in bold.

**(A)**

Response: **Hexanoic acid** Chisq Df Pr(>Chisq)

**GENOTYPE 68.3929 1 <2e-16 *****

SEX 0.7159 1 0.3975

FEED_RESTRICTION 0.0142 1 0.9051

GENOTYPE:SEX 0.0146 1 0.9038

SEX:FEED_RESTRICTION 0.2112 1 0.6458

GENOTYPE: FEED_RESTRICTION 1.4062 1 0.2357

**(B)**
Response: **Decane_3,8_dimethyl** Chisq Df Pr(>Chisq)

**GENOTYPE 204.1951 1 < 2e-16 *****

SEX 0.0145 1 0.90427

FEED_RESTRICTION 2.4961 1 0.11413

GENOTYPE:SEX 0.1371 1 0.71122

SEX:FEED_RESTRICTION 0.9025 1 0.34212

**GENOTYPE:FEED_RESTRICTION**  **4.1143 1 0.04252 ***

**(C)**

Response: **Decane_2,3,4_dimethyl** Chisq Df Pr(>Chisq)

**GENOTYPE 83.0297 1 < 2e-16 *****

SEX 0.0166 1 0.89739

FEED_RESTRICTION 0.2216 1 0.63786

GENOTYPE:SEX 0.3983 1 0.52797

SEX:FEED_RESTRICTION 2.9444 1 0.08617 .

GENOTYPE:FEED_RESTRICTION 3.0912 1 0.07872 .

**(D)**

Response: **1_Penten_3_ol** Chisq Df Pr(>Chisq)

**GENOTYPE 24.0613 1 9.332e-07 *****

SEX 0.4944 1 0.4820

FEED_RESTRICTION 0.1210 1 0.7279

GENOTYPE:SEX 2.2016 1 0.1379

SEX:FEED_RESTRICTION 1.5603 1 0.2116

GENOTYPE:FEED_RESTRICTION 0.1638 1 0.6856

**(E)**

Response: **Undecane**  Chisq Df Pr(>Chisq)

**GENOTYPE 73.9413 1 < 2e-16 *****

SEX 0.1173 1 0.73202

FEED_RESTRICTION 0.3969 1 0.52871

GENOTYPE:SEX 0.3016 1 0.58287

SEX:FEED_RESTRICTION 2.1419 1 0.14332

**GENOTYPE:FEED_RESTRICTION** **4.1075 1 0.04269**

**(F)**

Response:**3_Ethyl_3_methylheptane**

Chisq Df Pr(>Chisq)

GENOTYPE 1.5698 1 0.21023

SEX 2.4321 1 0.11887

FEED_RESTRICTION 0.5551 1 0.45623

GENOTYPE:SEX 2.8824 1 0.08955 .

SEX:FEED_RESTRICTION 2.6329 1 0.10467

GENOTYPE:FEED_RESTRICTION 0.0029 1 0.95678

**(G)**

Response: **Butanal_3_methyl** Chisq Df Pr(>Chisq)

**GENOTYPE 26.5004 1 2.635e-07 *****

**SEX 5.4803 1 0.01923 ***

FEED_RESTRICTION 0.2201 1 0.63897

GENOTYPE:SEX 0.0751 1 0.78408

SEX:FEED_RESTRICTION 0.3608 1 0.54809

GENOTYPE:FEED_RESTRICTION 0.0720 1 0.78849

**(H)**

Response: **Undecane_2_methyl** Chisq Df Pr(>Chisq)

**GENOTYPE 234.0734 1 < 2e-16 *****

SEX 0.0205 1 0.88627

FEED_RESTRICTION 0.8455 1 0.35783

GENOTYPE:SEX 0.0157 1 0.90030

SEX:FEED_RESTRICTION 1.3190 1 0.25077

GENOTYPE:FEED_RESTRICTION 3.4817 1 0.06205

**(CH)**

Response: **Hexanal**

Chisq Df Pr(>Chisq)

GENOTYPE 2.6260 1 0.1051

SEX 2.1191 1 0.1455

FEED_RESTRICTION 0.4747 1 0.4908

GENOTYPE:SEX 1.0787 1 0.2990

SEX:FEED_RESTRICTION 0.5169 1 0.4722

GENOTYPE:FEED_RESTRICTION 0.8088 1 0.3685

**(I)**

Response: **2,2,11,11-tetramethyldodecane**

Chisq Df Pr(>Chisq)

GENOTYPE 0.7398 1 0.3897

SEX 2.3368 1 0.1263

FEED_RESTRICTION 0.0712 1 0.7896

GENOTYPE:SEX 2.0693 1 0.1503

SEX:FEED_RESTRICTION 1.5679 1 0.2105

GENOTYPE:FEED_RESTRICTION 0.0347 1 0.8521
